# Anti-Inflammatory, Cytotoxic, and Genotoxic Effects of Soybean Oligopeptides Conjugated with Mannose

**DOI:** 10.3390/foods13162558

**Published:** 2024-08-16

**Authors:** Pornsiri Pitchakarn, Pensiri Buacheen, Sirinya Taya, Jirarat Karinchai, Piya Temviriyanukul, Woorawee Inthachat, Supakit Chaipoot, Pairote Wiriyacharee, Rewat Phongphisutthinant, Sakaewan Ounjaijean, Kongsak Boonyapranai

**Affiliations:** 1 Department of Biochemistry, Faculty of Medicine, Chiang Mai University, Muang Chiang Mai, Chiang Mai 50200, Thailand; pornsiri.p@cmu.ac.th (P.P.); pensiri.bua@cmu.ac.th (P.B.); jirarat.ka@cmu.ac.th (J.K.); 2 Multidisciplinary Research Institute, Chiang Mai University, Chiang Mai 50200, Thailand; sirinya.t@cmu.ac.th (S.T.); supakit.ch@cmu.ac.th (S.C.); rewat.p@cmu.ac.th (R.P.); 3 Institute of Nutrition, Mahidol University, Salaya, Nakhon Pathom 73170, Thailand; piya.tem@mahidol.ac.th (P.T.); woorawee.int@mahidol.ac.th (W.I.); 4 Processing and Product Development Factory, The Royal Project Foundation, Chiang Mai 50100, Thailand; pairote.w@cmu.ac.th; 5 Research Institute for Health Sciences, Chiang Mai University, Chiang Mai 50200, Thailand; sakaewan.o@cmu.ac.th

**Keywords:** soybean, conjugated peptide, biological activity, safety assessment, mutagenicity, wing spot test

## Abstract

Soy protein is considered to be a high-quality protein with a range of important biological functions. However, the applications of soy protein are limited due to its poor solubility and high level of allergenicity. Its peptides have been of interest because they exert the same biological functions as soy protein, but are easier to absorb, more stable and soluble, and have a lower allergenicity. Moreover, recent research found that an attachment of chemical moieties to peptides could improve their properties including their biodistribution, pharmacokinetic, and biological activities with lower toxicity. This study therefore aimed to acquire scientific evidence to support the further application and safe use of the soybean oligopeptide (OT) conjugated with allulose (OT-AL) or D-mannose (OT-Man). The anti-inflammation, cytotoxicity, and genotoxicity of OT, OT-AL, and OT-Man were investigated. The results showed that OT, AL, Man, OT-AL, and OT-Man at doses of up to 1000 µg/mL were not toxic to HepG2 (liver cancer cells), HEK293 (kidney cells), LX-2 (hepatic stellate cells), and pre- and mature-3T3-L1 (fibroblasts and adipocytes, respectively), while slightly delaying the proliferation of RAW 264.7 cells (macrophages) at high doses. In addition, the oligopeptides at up to 800 µg/mL were not toxic to isolated human peripheral blood mononuclear cells (PBMCs) and did not induce hemolysis in human red blood cells (RBCs). OT-Man (200 and 400 µg/mL), but not OT, AL, Man, and OT-AL, significantly reduced the production of NO and the expression of inducible nitric oxide synthase (iNOS) and cyclooxygenase-2 (COX2) stimulated by lipopolysaccharide (LPS) in RAW 264.7 cells, suggesting that the mannose conjugation of soy peptide had an inhibitory effect against LPS-stimulated inflammation. In addition, the secretion of interleukin-6 (IL-6) stimulated by LPS was significantly reduced by OT-AL (200 and 400 µg/mL) and OT-Man (400 µg/mL). The tumor necrosis factor-α (TNF-α) level was significantly decreased by OT (400 µg/mL), AL (400 µg/mL), OT-AL (200 µg/mL), and OT-Man (200 and 400 µg/mL) in the LPS-stimulated cells. The conjugation of the peptides with either AL or Man is likely to be enhance the anti-inflammation ability to inhibit the secretion of cytokines. As OT-Man exhibited a high potential to inhibit LPS-induced inflammation in macrophages, its mutagenicity ability was then assessed in bacteria and *Drosophila*. These findings showed that OT-Man did not trigger DNA mutations and was genome-safe. This study provides possible insights into the health advantages and safe use of conjugated soybean peptides.

## 1. Introduction

Soybean (*Glycine max*) is a type of legume, classified as the family Leguminosae, and can be processed into various products such as soymilk, tofu, tempeh, or sprouts. Soybean is rich in nutrients with a protein content of 35–40%, depending on the variety and the location and climate of the planting [1,2]. It also has a carbohydrate and lipid content of approximately 23% and 20%, respectively [1,2]. Soybean protein is considered to be a high-quality protein because it contains twenty types of amino acids which include nine essential amino acids that are equivalent to those found in animal protein [3,4]. In addition, it has been reported that soy proteins and peptides exert many health promotion advantages against obesity [5], cardiovascular disease [6], insulin-resistance/type II diabetes [7], certain type of cancers [8], and immune disorders [9]. However, soy protein has a poor solubility and high allergenicity [10,11]. Soybean peptides are produced from the proteolytic hydrolysis of soy protein and consist of 3–6 amino acids as a mixture of oligopeptides with a molecular weight below 1000 Da [11,12]. Soy peptides not only exert the same biological functions as soy protein, but they are also easier to absorb, more stable and soluble, and have a lower allergenicity [10,12,13]. Accordingly, soy peptides have become of interest as functional foods which play roles in the modulation of physiological functions or the prevention of chronic diseases such as hypolipidemia and hypertension and can also show anti-cancer properties and anti-inflammatory, antioxidant, and immunomodulatory effects [14]. The attachment of chemical moieties to peptides or conjugation with peptides has been studied to improve their properties including their biodistribution, pharmacokinetic, toxicity, and biological activities [15]. Biological molecules such as lipids, sugars, and proteins can be used as conjugates with various properties. It was previously demonstrated that the conjugation of lauric acid with antimicrobial peptides enhanced their ability to form secondary structures upon interacting with bacterial membranes [16]. α-lactalbumin and its hydrolysate conjugated with a rare sugar, d-allulose or d-psicose, have significantly enhanced antioxidant properties [17]. Allulose has also been reported for its abilities to improve glycemic control and reduce adiposity and prevent metabolic disorders [18,19]. In addition, mannosylation has been reported as an efficient strategy to improve the uptake and targeting ability of drugs or interested compounds to mannose receptor-expressing cells, including macrophages. Recently, mannose is acquiring more interest as a promising target ligand in targeted therapy, especially cancer therapy [20].

In addition, soy protein hydrolysate conjugated with curdlan polysaccharide showed excellent gel-forming and emulsifying properties in food processing and exhibited a higher antioxidant activity than the unconjugated form [21]. Additionally, several previous studies of soy protein conjugated with mono/polysaccharides showed enhancements in a range of functional characteristics, such as sensory attributes, solubility, thermal stability, structural flexibility, freeze–thaw stability, hydrophilicity, and antioxidant activity [21,22,23,24,25,26,27,28]. In our previous study, we formulated soy protein isolate hydrolysate–yeast cell extract (SPIH-YCE) conjugates through a humid–dry heating process. The conjugates demonstrated higher bioactive activities including antioxidant, acetylcholinesterase (ACE) inhibitory, and anti-inflammatory activities than the unconjugated peptides [25]. This finding may be due to the Maillard reaction between oligopeptides (OT) and monosaccharides, which could produce advance glycation end-products (AGEs). This distributed to the change in OT conformation such as increased the formation of new functional groups, and the OT biological function including a stronger antioxidant property as AGEs can scavenge free radicals more efficiently than their precursor peptides.

In this study, we intensively explore the anti-inflammatory activity of the oligopeptide (OT) conjugated with allulose (OT-AL) and D-mannose (OT-Man) in lipopolysaccharide (LPS)-induced RAW 264.7 macrophages. The LPS-induced inflammation model is widely employed to determine the anti-inflammatory potential of test compounds [26]. LPS could stimulate inflammatory-related signaling including the nuclear factor kappa-light-chain-enhancer of activated B cells (NF-κB) pathways. The activation of NF-κB induces the expression of pro-inflammatory cytokines and mediators such as tumor necrosis factor (TNF)-α, interleukin (IL)-1β, IL-6, inducible nitric oxide synthase (iNOS), and cyclooxygenase (COX)-2. The expression of iNOS and COX-2 causes the overproduction of nitric oxide (NO), reactive oxygen species (ROS), and prostaglandin E2 (PGE2), key molecules in the inflammation process [26,27]. Furthermore, the safety of the monosaccharide-conjugated oligopeptides was investigated using cytotoxicity and genotoxicity assessments.

Toxicity testing is an important part of assessing the potential adverse effects of a test substance, including plant extracts or biological active compounds [28]. Cytotoxicity testing is a screening tool and an initial step for toxicity assessment. Several mouse- and human-based cell lines have been used for general cytotoxicity testing. These include mouse adipocyte fibroblasts (3T3-L1), mouse macrophage cells (RAW 264.7), human lung fibroblasts (MRC-5), human embryonic kidney cells (HEK293), human hepatic stellate cells (LX-2), human colon carcinoma cells (Caco2), and human hepatocellular carcinoma cells (HepG2), as well as human peripheral blood mononuclear cells (hPBMCs) and human red blood cells (hRBCs), to represent target organs and blood circulation in humans [29,30,31,32]. In addition, cytotoxicity testing can also be used to estimate starting doses for acute oral systematic toxicity tests in animal models [33]. Genotoxicity testing is another crucial step in investigating the ability of substances of interest to damage genetic information in organisms [34]. Being exposed to chemicals or biological agents can result in genomic instabilities and/or epigenetic alterations, resulting in mutagenesis leading to cancer [35]. Therefore, in the risk assessment of substances of interest, their potential mutagenicity must be considered. The bacterial reverse mutation assay, or the Ames test, is the most successful and widely used test as an initial screening process to determine the potential mutagenic activity of a substance. It is based on testing the capacity of the substrate to revert mutations in the tester mutant bacteria that restore its ability to synthesize an essential amino acid required for growth [36]. Some substances are pro-mutagens, requiring metabolic activation to be active mutagens; therefore, mouse liver homogenate, the S9 mixture, is applied as a metabolic activator to mimic mutagenesis via CYP450 metabolism [37]. The standard three-test battery recommendations include an assessment for gene mutation in bacteria, chromosomal damage in mammalian cells, or a mouse lymphoma thymidine kinase (Tk+/−) assay (MLA) and chromosomal damage of hematopoietic cells in rodents [38]. In addition to the Ames test, mutagenicity assessment using the somatic mutation and recombination test (SMART) or the wing spot test in *Drosophila melanogaster* is an effective in vivo alternative model [39,40]. The SMART model for food safety testing is well-known and helpful due to the short life cycle, large numbers of embryos, and easy genetic modification of *Drosophila*. This technique can be used to detect wide ranges of DNA damage, including point mutations and DNA breaks, as well as mitotic recombination [39]. Additionally, *D. melanogaster* can activate procarcinogens and mutagens enzymatically in vivo through endogenous cytochrome P450 (CYP) mono-oxygenases-dependent activation systems [40].

This study investigated the comparison between anti-inflammation allulose-conjugated OT (OT-AL) and D-mannose-conjugated OT (OT-Man) with unconjugated OT. The anti-inflammatory activity of the compounds was measured in an LPS-induced RAW 264.7 macrophage model. The aim was to see whether the conjugation could enhance the anti-inflammation ability of OT. The toxicity of OT-AL and OT-Man was also investigated using cytotoxicity testing in HepG2, HEK293, LX-2, and RAW 264.7 cells, pre- and mature 3T3-L1 adipocytes, and also in isolated human primary cells, human peripheral blood mononuclear cells (PBMCs), and red blood cell (RBCs). In addition, the mutagenicity of the conjugated oligopeptides was assessed using the Ames test and SMART. The results from this study will be helpful in informing further investigations and also support the safe use of the conjugated soybean peptides as ingredients in functional foods.

## 2. Materials and Methods

### 2.1. Chemicals and Reagents

Soy protein isolate (~87% dry basis of protein content from Food Great Products Co., Ltd., Bangkok, Thailand) was used as the raw material in this study. The Alcalase enzyme from *Bacillus licheniformis* (>0.75 AU/mL) was purchased from Merck (Darmstadt, Germany). Sugar standards including D-allulose and D-mannose were purchased from Sigma-Aldrich (Singapore). 3-isobutyl-1-methylxanthine (IBMX), dexamethasone (DEX), the Griess reagent, insulin, lipopolysaccharide (LPS), and sulphorhodamine B (SRB) were purchased from Sigma-Aldrich (Darmstadt, Germany). Ficoll Hypaque (Lymphoprep™) was purchased from STEMCWLL™ Technologies (Vancouver, BC, Canada).

### 2.2. Preparation and Characterization of Soybean Oligopeptide (OT), Allulose-Conjugated OT (OT-AL), and D-Mannose-Conjugated OT (OT-Man)

Oligopeptide conjugates with mannose or allulose were prepared using a slightly modified methodology based on Phongphisutthinant et al. [25]. First, soybean oligopeptide (OT) was produced by mixing soybean flour (SF) with deionized water (DI) at a 1:5 (*w*/*v*) ratio and agitating until homogeneous. Soybean protein was solubilized by adjusting the pH to 8–9 using 5 M NaOH and stirring for 1 h. After centrifugation at 7000× *g* for 5 min, the soluble protein in the supernatant was precipitated by adjusting the pH to 5.0 with 5 M HCl. The precipitated protein was desalted using a 10 kDa dialysis tube for 72 h, followed by re-centrifugation and freeze-drying. The resulting soy protein isolate was defatted with hexane and stored at −5 °C. Next, soy protein hydrolysate was prepared by dispersing 5 g of soy protein isolate in deionized water at a 1:40 (*w*/*v*) ratio. This mixture was combined with the Alcalase enzyme at an enzyme-to-substrate ratio of 1% (*v*/*w* protein) and agitated gently at 50 °C for 10 h. The reaction was stopped by heating the mixture in boiling water (95 ± 2 °C) for 15 min, followed by cooling. The clear supernatant was freeze-dried to obtain soybean oligopeptide (OT), which was then stored at 5 °C until further use in the conjugation process.

For the conjugation process, OT was prepared at a concentration of 1% (*w*/*v*) in deionized water and then combined in a 1:1 (*v*/*v*) ratio with 1% (*w*/*v*) solutions of mannose or allulose, referred to as OT-Man or OT-AL, respectively. The mixtures were then freeze-dried and incubated at 60 °C with 75% relative humidity for 10 days, controlled by a saturated NaCl solution. The resulting oligopeptide conjugates were stored at 5 °C for further analysis. The chemical characteristics, composition of amino acids contained in OT, and molecular weight (MW) of OT, OT-Man, and OT-AL were measured according to our previous study [25]. The protein content of OT was 58 g/100 g, while the composition of amino acids predominantly found in OT (100 g) was proline (45 mg), lysine (22 mg), phenylalanine (11 mg), histidine (9 mg), and tyrosine (7 mg) as reported in our previous study [25]. In addition, size exclusion analysis by HPLC found that the MW of OT was in the range of > 0.1 kDa, 0.10–1.0 kDa, and 1.0–10.0 kDa at approximately 5%, 45%, and 50%, respectively. After the conjugation process, the MW of OT-Man was in the range of >0.1 kDa, 0.10–1.0 kDa, and 1.0–10.0 kDa at approximately 7%, 4%, and 89%, respectively, while the MW of OT-AL was in the range of >0.1 kDa, 0.10–1.0 kDa, and 1.0–10.0 kDa at approximately 3%, 6%, and 91%, respectively. These may represent the success of the conjugation. The achievement of the conjugation of OT with either Man or AL was further confirmed by SDS-PAGE [25]. The increase in peptide MW was evidenced by the presence of polydisperse bands in the middle to the top of the SDS-PAGE gel. This can be attributed to glycosylation between peptides and the monosaccharide, suggesting the formation of conjugated compounds [25].

### 2.3. Cell Culture

HEK293 (Human embryonic kidney cell line), HepG2 (Human liver carcinoma cell line), LX-2 (Human hepatic stellate cell line), and 3T3-L1 (Murine preadipocyte cell line) cells were obtained from the American Type Culture Collection (ATCC), Bethesda, MD, USA. The cells were cultured in Dulbecco’s Modified Eagle Medium (DMEM) (Gibco™, Fisher Scientific, Loughborough, UK) with L-glutamine supplemented with 10% heat-inactivated fetal bovine serum (FBS, Sigma, St. Louis, MO, USA) for HepG2, HEK293, and LX2 or 10% heat-inactivated calf serum (CS, Gibco™, Fisher Scientific) for 3T3-L1 and 1% penicillin/streptomycin solution and maintained at 37 °C in a 5% CO_2_ humidified atmosphere (CO_2_ incubator, Thermo Scientific, Waltham, MA, USA) and sub-cultured every 3 days.

The pre-adipocyte 3T3-L1 cell line was differentiated to become mature adipocytes by culturing the cells to 100% confluence in 10% calf serum DMEM (CS medium), as Day 0 in a 96 well-plate. After post-confluence, the cells were incubated with the induction medium as a differentiation initiation medium (DMEM with or without 1 μM DEX, 0.5 mM IBMX, 167 nM insulin, and 10% FBS) for 3 days (Days 1–3). After that, cells were cultured in a differentiation medium as an adipogenic differentiation medium (differentiation media: DMEM containing 167 nM insulin and 10% FBS) for 3 days (Days 4–6). Finally, maturation was induced in an induced-lipid accumulation medium (maturation media: DMEM containing 167 nM insulin and 10% FBS) for 6 days (Days 7–12). The mature adipocytes were then used for toxicity testing.

A murine macrophage cell line RAW 264.7 obtained from CLS-Cell Lines Service, Eppelheim, Germany, was cultured in Dulbecco’s Modified Eagle Medium (DMEM, Sigma, St. Louis, MO, USA) in the presence of L-glutamine, 1% penicillin/streptomycin (FBS, Sigma, St. Louis, MO, USA), and 10% heat-inactivated fetal bovine serum (FBS, Sigma, St. Louis, MO, USA) in an ultra-low binding culture dish (Corning^®^, Oneonta, NY, USA). The cells were maintained in a 5% CO_2_ humidified atmosphere (CO_2_ incubator, Thermo Scientific, Waltham, MA, USA) at 37 °C and sub-cultured every 3 days.

As described by Karinchai et al., human peripheral blood mononuclear cells (PBMCs) and red blood cells (RBCs) were isolated from human blood samples [41]. Human blood samples of healthy subjects obtained from Maharaj Hospital, Chiang Mai, Thailand, were anonymized and kept in a heparinized tube by the laboratory. RBCs were collected and used to determined hemolysis induction by a hemolysis assay. Ficoll-Hypaque solution was used to isolate PBMCs, as described in the manufacturer’s instructions [42]. A certificate of exemption by the Research Ethics Committee of the Faculty of Medicine, Chiang Mai University (No. EXEMPTION 8989/2022), was granted. Consent to participate was not applicable as all data were anonymized. All methods were carried out in accordance with relevant guidelines and regulations.

### 2.4. Cytotoxicity Assay

The cytotoxicity of the oligopeptide and its conjugated forms on HepG2, HEK293, LX-2, and RAW 264.7 cells, pre- and mature 3T3-L1 adipocytes, and hPBMCs was analyzed by a sulforhodamine (SRB) assay [43] to obtain their non-toxic concentration for further experiments and as screening data for their in vitro safety. The cells were plated for 12 h in 96-well plates and then treated with the oligopeptide and its conjugated sugar at a concentration of 0–1000 µg/mL for 48 h. Dimethyl sulfoxide (DMSO) was used as a vehicle control (the untreated control). At the indicated time, the cells were then fixed and subjected to an SRB assay. The absorbance was measured at 510 nm by spectrophotometry (Gen5, BioTek, Winooski, VT, USA), and the cell viability was calculated as the ratio of the treatment versus untreated control. The data are represented as the mean ± standard deviation of three replicates and are expressed relative to the untreated control. The percentage of cell survival was calculated as follows:

% cell survival = (Abs_extract/Abs_control) × 100

### 2.5. Hemolysis Assay

The hemolytic effect of the oligopeptide and its conjugated forms was determined by hemolysis assay. Briefly, the 5% RBCs suspension was mixed with various concentrations of the samples and further incubated at 37 °C for 3 h. The hemolysis induction by the compounds was measured as described previously [44]. The hemolytic effect of each compound was interpreted using the following guidelines: 0–10% hemolysis is non-hemolysis, 10–25% hemolysis is slight hemolysis induction, and >25% hemolysis is high hemolysis induction [45].

### 2.6. Measurement of Nitric Oxide (NO)/Nitrite 

To screen the anti-inflammation ability of the compounds, the nitrite level in the cultured medium of the treated cells was determined by the Griess reaction [46]. RAW 264.7 macrophage cells were pretreated with various concentrations of the oligopeptide and its conjugated forms (100, 200, 400 µg/mL) for an hour and then incubated with LPS (1 µg/mL) for further 24 h at 37 °C in a 5% CO_2_ humidified atmosphere. The culture supernatant was collected for the Griess reagent assay as previous described [47]. The NO production was calculated by comparison with LPS-treated controls.

### 2.7. Pro-Inflammatory Cytokine Determination

RAW 264.7 macrophage cells were pretreated with various concentrations of the oligopeptide and its conjugated forms (100, 200, 400 µg/mL) for an hour and then incubated with LPS (1 µg/mL) for further 24 h at 37 °C in a 5% CO_2_ humidified atmosphere. Then, a sandwich Enzyme Link Immuno-Sorbent Assay was carried out in accordance with the manufacturer’s protocols (BioLegend ELISA MAXTM Deluxe Set, San Diego, CA, USA) to measure TNF-α or IL-6 protein levels in the condition medium.

### 2.8. Immunoblotting

RAW 264.7 macrophage cells were pretreated with various concentrations of the oligopeptide and its conjugated forms (100, 200, 400 µg/mL) for an hour and then incubated with LPS (1 µg/mL) for a further 24 h (at 37 °C in a 5% CO_2_ humidified atmosphere). The cells were then collected to investigate the iNOS and COX-2 protein levels. Cell lysate was prepared to obtain the protein samples. The samples were subjected to 10% sodium dodecyl sulfate polyacrylamide gel electrophoresis (SDS-PAGE). The separated proteins were electrically transferred to a nitrocellulose membrane. The target proteins were probed with specific antibodies, including anti-COX-2 antibody (Cell signaling, Danvers, MA, USA), anti-iNOS antibody (Merck, Rahway, NJ, USA), and anti-β-actin antibody (Sigma, St. Louis, MO, USA). Protein detection was performed using a chemiluminescence reagent (Bio-Rad Laboratories, Watford, UK).

### 2.9. Mutagenicity Testing of the Oligopeptide and Its Mannose-Conjugated Form Using the Salmonella Mutation Assay

The mutagenicity of OT, Man, and OT-Man was performed using the *Salmonella typhimurium* strains TA98 and TA100, with or without metabolic activation (S9 mix) [48]. Overnight bacterial cultures were mixed with various doses of test compounds with (+S9) or without (−S9) and incubated at 37 °C for 20 min. The top agar was added and then poured onto a plate of minimal glucose agar. The plates were incubated at 37 °C for 48 h. The number of revertant colonies was counted and the mutagenic index (MI) was calculated. 2-aminoanthracene (2-AA) and 2-(2-furyl)-3-(5-nitro-2-furyl)-acrylamide (AF-2) were used as positive mutagens in the presence and absence of S9.

### 2.10. In Vivo Genotoxicity Evaluation of the Oligopeptide and Its Mannose-Conjugated Form Using the Somatic Mutation and Recombination Test (SMART) in Drosophila melanogaster

The in vivo wing spot test or SMART involving *Drosophila melanogaster* was used to test the mutagenic potential of Man, OT, and OT-Man. In brief, two strains of fruit flies, including *mwh*/*mwh* and ORR; *flr^3^/In(3LR) TM3, ri p^p^ sep l(3)89Aa bx^34e^ e Bd^S^*, were mated. Following this, the third instar larvae, which were collected from the mating and harbor *mwh flr*+/*mwh TM*, were fed with a medium containing (i) deionized water (DI, negative control), (ii) 20 mM urethane (positive control), (iii) D-mannose (62.5–5000 µg/mL), and (iv) OT (62.5–5000 µg/mL) and OT-DM (62.5–5000 µg/mL). After hatching, forty round wings were collected and analyzed for mutant spots (single, large, and twin spots). The statistical analysis for the wing spot test was performed as previously described [49]. The *Drosophila* study was approved by the Institute of Nutrition—Mahidol University Institutional Animal Care and Use Committee (INMU-IACUC) (COA. No. INMU-IACUC 2023/01).

### 2.11. Statistical Analysis

All values are provided as the mean ± standard derivation (SD) from three independent experiments (triplicate samples). Overall differences between the treatment groups were analyzed using a one-way analysis of variance (ANOVA), followed by Tukey’s multiple comparison test using GraphPad Prism 9.4.1 software. A *p* value < 0.05 was considered statistically significant.

## 3. Results

### 3.1. Cytotoxicity of the Oligopeptide and Its Conjugated Forms

To investigate whether the oligopeptide and its conjugated forms affect cell viability, HepG2, HEK293, LX-2, pre- and mature-3T3-L1, and RAW 264.7 cells were treated with the peptides (0–1000 µg/mL) for 48 h. The results showed that OT, AL, Man, OT-AL, and OT-Man at up to 1000 µg/mL were not toxic to HepG2, HEK293, LX-2, and pre- and mature-3T3-L1 cells (Table 1), while the cell viability of RAW 264.7 cells was decreased by approximately 20% when the cells were treated with OT, OT-AL, and OT-Man at 60, 270, and 450 µg/mL, respectively, as shown in Table 1 and Table 2. OT obviously decreased the cell number of RAW 264.7 with an IC50 at approximately 510 µg/mL (Table 1). Microscopic observation revealed that the reduction in cell number caused by OT (Table 1) was due to the inhibition of cell proliferation rather than the induction of cell death. In addition, OT, AL, Man, OT-AL, and OT-Man at up to 800 µg/mL did not show any toxic effects to hPBMCs and did not induce hemolysis in hRBCs, as shown in Table 3 and Table 4. Hence, the concentration of OT, OT-AL, and OT-Man in the range of 0–400 µg/mL was used for the subsequent studies in this study.

### 3.2. The Effect of the Oligopeptide and Its Conjugated Forms on Nitric Oxide (NO) Production in LPS-Treated Macrophages

**Figure 1:**
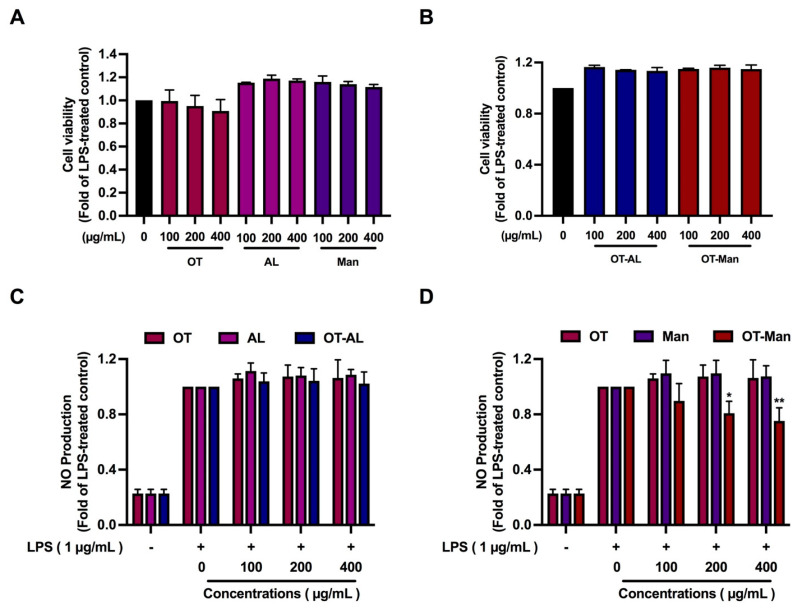
Anti-inflammation property of soybean oligopeptides (OT), allulose (AL), D-mannose (Man), and the oligopeptides conjugated with allulose (OT-AL) or D-mannose (OT-Man). Cytotoxicity of OT, AL, and Man (**A**) and the conjugated oligopeptides OT-AL and OT-Man (**B**) in murine macrophage RAW 264.7 cells in the presence of LPS (1 µg/mL for 24 h). The anti-inflammatory activities of OT, AL, and OT-AL (**C**) and Man, and OT-Man (**D**) in LPS-treated RAW 264.7 cells. Cell viability was measured after the cells were incubated in the presence of various concentrations of OT, AL, Man, OT-AL, and OT-Man for 24 h by SRB assay. The effect of OT, AL, Man, OT-AL, and OT-Man on LPS-induced nitric oxide (NO) production was determined by treating the cells with OT, AL, Man, OT-AL, or OT-Man for 1 h, and then 1 μg/mL of LPS was added and further incubation took place for 24 h. After that, the culture medium was collected and NO production was measured by the Griess reagent. The data are shown as the mean ± SD of three independent experiments. * *p* < 0.05 and ** *p* < 0.01 against LPS-treated control (without the compounds) on anti-inflammation.

Measurement of the nitrite level or nitric oxide (NO) production in the culture medium of LPS-treated macrophages can be used as an inflammatory screening marker to examine the anti-inflammatory ability of bioactive compounds. In the presence of LPS, OT, AL, Man, OT-AL, and OT-Man at up to 400 µg/mL the cell viability of RAW 264.7 cells did not change as shown in Figure 1A,B. This result could confirm the previous finding of the inhibitory effect of OT on cell proliferation.

OT, AL, Man, and OT-AL did not inhibit LPS-induced NO production, while OT-Man at 200 and 400 µg/mL significantly reduced NO production by 20% and 25%, respectively (Figure 1C,D), highlighting the effects of mannose conjugation on the inhibitory effect of soy peptide against LPS-stimulated NO production.

### 3.3. Effect of the Oligopeptide and Its Conjugated Forms on iNOS and COX-2 Levels in LPS-Treated RAW 264.7 Cells

**Figure 2:**
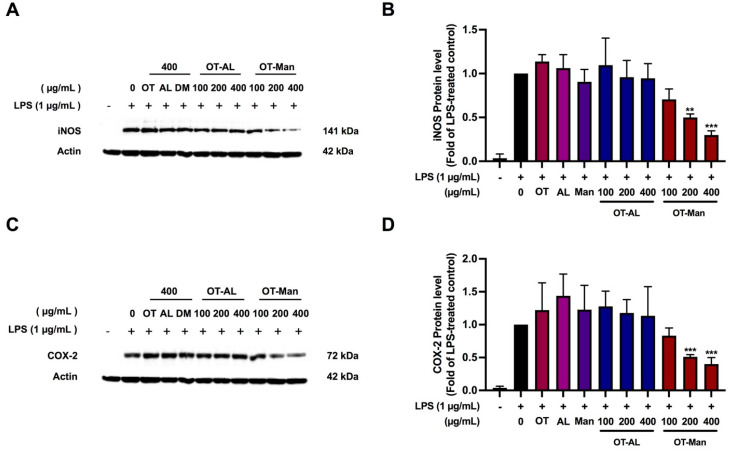
Effects of oligopeptides (OT), allulose (AL), D-mannose (Man), and the oligopeptides conjugated with allulose (OT-AL) and D-mannose (OT-Man) on LPS-induced iNOS (**A**,**B**) and COX-2 (**C**,**D**) protein level. After the treatment, the protein samples were collected and used to determine the iNOS and COX-2 levels by Western blotting (normalized with β-actin level). The data are shown as the mean ± SD of three independent experiments. ** *p* < 0.01 and *** *p* < 0.001, against LPS-treated control (without the compounds).

NO production is based on the activity of inducible nitric oxide synthase (iNOS), while cyclooxygenase-2 (COX-2) is involved in inflammatory responses by producing prostaglandin E2 (PGE2); thus, we also measured the protein level of iNOS and COX-2 to molecularly explain the results shown in Figure 1. Figure 2 shows that the expression of iNOS and COX-2 was markedly upregulated in LPS-treated macrophages, while their levels of expression were reduced by 50% and approximately 60–70% when the LPS-stimulated cells were treated with OT-Man at 200 and 400 mg/mL, respectively (Figure 2). OT, AL, Man, and OT-AL did not affect the iNOS and COX-2 protein levels. The results suggest that LPS-induced NO production was inhibited by OT-Man via a reduction in iNOS protein expression. The suppression of the COX-2 protein level by OT-Man predicted the decrease in PGE2 production. The mechanism used by OT-Man to inhibit inflammation could occur via the suppression of LPS-stimulated iNOS and COX-2 levels. However, other secretory proteins are also able to induce inflammation or chemotaxis. Thus, whether the peptides could inhibit the secretion of the pro-inflammatory cytokines, TNF-α and IL-6 induced by LPS was further evaluated in the next experiment.

### 3.4. Effect of OT-AL and OT-Man on the Secretion of IL-6 and TNF-α in LPS-Treated RAW 264.7 Cells

**Figure 3:**
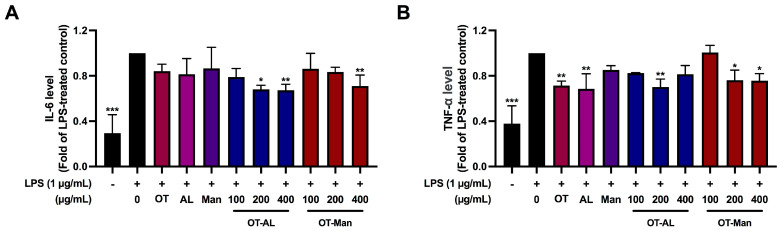
Effects of oligopeptides (OT), allulose (AL), D-mannose (Man), and the oligopeptides conjugated with allulose (OT-AL) and D-mannose (OT-Man) on LPS-induced IL-6 (**A**) and TNF-α (**B**) secretions in the murine macrophages. The culture medium was collected after pre-treatment of the cells with the compounds for 1 h followed by further co-treatment with LPS (1 µg/mL) for 24 h. IL-6 and TNF-α levels in the culture medium were determined by ELISA. The data are shown as the mean ± SD of three independent experiments. * *p* < 0.05, ** *p* < 0.01, and *** *p* < 0.001, against LPS-treated control (without the compounds).

As shown in Figure 3, LPS treatment markedly induced the secretion of IL-6 and TNF-α. The secretion of IL-6 stimulated by LPS was significantly reduced at approximately 30% by OT-AL (200 and 400 µg/mL) and OT-Man (400 µg/mL) (Figure 3A). OT, AL, and Man did not alter the level of IL-6. Figure 3B shows that the TNF-α level was significantly decreased by OT (400 µg/mL), AL (400 µg/mL), OT-AL (200 µg/mL), and OT-Man (200 and 400 µg/mL) in the LPS-stimulated cells. The results are consistent with previous reports which found that soy peptides and allulose possessed anti-inflammatory action [50,51].

Taken together, the conjugation of the peptides with either AL or Man is likely to enhance the level of anti-inflammation ability to inhibit the secretion of the cytokines.

### 3.5. Mutagenicity of Oligopeptide (OT), D-Mannose (Man), and Mannose Conjugated Oligopeptide (OT-Man)

The study into the levels of anti-inflammation revealed that OT-Man exhibits a higher potential to inhibit LPS-induced inflammation in macrophages. This study therefore determined the genotoxicity of OT, Man, and OT-Man and found that the compounds did not cause any mutagenicity in the *S. typhimurium* strains TA98 and TA100 in the presence and absence of the S9 mix (Table 5). However, the highest dose (5 mg/plate) of OT had a killing effect on bacteria of the strain TA98, whereas OT and OT-Man had a killing effect on the strain TA100 in the presence and absence of the S9 mix.

### 3.6. In Vivo Genotoxicity Analysis in Drosophila Using SMART

In addition to the in vitro genotoxicity test (Table 5), the in vivo wing spot test in *Drosophila* was subsequently utilized to investigate the mutagenic potential of D-mannose, OT and OT-DM. The advantages of SMART are as follows: (i) it is a short-term test that employs a bioactivation system that similar to the human biotransformation system, which is characterized by the expression of cytochrome P450 [39], and (ii) this assay can detect various types of DNA damage, such as point mutations, frameshift mutations, and DNA or chromosome breaks [39]. The frequency of mutant spots per individual from forty fly wings is shown in Table 6. The flies in the negative control group exhibited an average total mutant spot of 0.68, while urethane-exposed flies (positive control) showed a clear display of high mutant spots categorized as single, large, and twin spots, culminating in a high total mutant spot of 10.18 (15-fold greater than the negative control). The data confirmed the mutagenic potential of urethane which was in agreement with previous reports [52,53]. The results in Table 6 also show that in all doses OT, Man, and OT-Man, even at the highest concentration at 5000 µg/mL, the single, large, twin, and total mutant spots were between 0.13 and 0.53, which was comparable with the negative control. This evidence implies that OT, Man, and OT-Man did not trigger DNA mutations and were genome-safe.

## 4. Discussion

Soy bioactive peptides, small protein fragments resulting from protein hydrolysis, have been of interest in the research and development of functional foods and human health-related products because of their advantageous properties over the entire protein form, including being easier to absorb, more stable and soluble, and having a lower allergenicity [9,10,12,13,54,55]. In addition, soy peptides exhibit various biological activities such as hypolipidemia, anti-hypertensive, and anti-cancer properties, and anti-inflammatory, antioxidant, and immunomodulatory effects [14]. Recently, much attention has been paid to the combination of two or more bioactive moieties in one molecule or formula which can be an effective strategy for the design and development of novel functional foods. For example, Li et al. demonstrated that the glycation of salmon protein with reducing sugars has been shown to enhance its anti-inflammatory activity [54]. Therefore, oligopeptide conjugation has emerged as a promising strategy for enhancing the biological activities of some proteins. Our previous study achieved the conjugation process of soy oligopeptide with yeast cell extract [25]. It was found that the conjugated form exhibits higher antioxidant, anti-inflammation, and ACE inhibitory activities than the unconjugated oligopeptide [25]. However, screening of the anti-inflammatory activity of the conjugated oligopeptides in our previous study was performed in vitro but not in a cell culture model which is more suitable and reliable. In this study, allulose-conjugated OT (OT-AL) and D-mannose-conjugated OT (OT-Man) were formulated and subjected to investigation of their anti-inflammation activity using LPS-stimulated macrophages as a model. According to the previous report, the humidity (75%), incubation time (10 days), and temperature (60 °C) during the conjugation process influence the degree of glycation and achievement of the process which may lead to an enhancement of the bioactivity of the conjugated OT [25]. The present study therefore prepared oligopeptides conjugated with Man or AL based on our previous study with a slight modification.

The production of nitric oxide (NO), an inflammatory maker, was measured for the screening of anti-inflammation properties [56]. The results found that only OT-Man significantly inhibit LPS-stimulated NO production. Consistent with this result, OT-Man significantly decreased the expression of iNOS, a key inducible enzyme producing NO, and COX-2, a key enzyme in the production of PGE2, indicating that OT-Man could inhibit LPS-induced inflammation through the suppression of the expression of key mediated enzymes, iNOS and COX-2. Mannose can selectively target specific cells or tissues, particularly immune cells, by interacting with mannose receptors. This targeted delivery mechanism not only enhances the effectiveness of therapeutic interventions but also facilitates immune modulation [57,58]. Chu et al. demonstrated that the density of mannose conjugated to polymeric nanoparticles impacted in vitro uptake and RNAi efficacy in RAW 264.7 cell polymeric nanoparticles [59]. In addition, mannose-decorated hybrid nanoparticles enhanced the targeting localized on the surface of macrophages and increased the nanoparticle uptake by macrophages [60,61]. As the macrophages express mannose receptors on the surface membrane [62], it might be possible that the conjugation of the oligopeptide with mannose could enhance the inflammation ability through increasing the cellular uptake of the oligopeptide. In addition, the overexpression of pro-inflammatory cytokines, such as IL-6, TNF-α, and IL-1β, from the macrophages is further involved in the upregulation of inflammatory reactions resulting in inflammation-related diseases [63,64]. Therefore, decreasing pro-inflammatory secretion could ameliorate several inflammation-related conditions. Our study demonstrated that IL-6 secretion was significantly decreased by the treatment of OT-AL and OT-Man. In addition, OT, AL, OT-AL, and OT-Man significantly suppressed the secretion of TNF-α. Wen et al. reported that soybean peptides could inhibit LPS-induced inflammation by reducing the production of TNF-α, IL-1β, and IL-6 [65]. Mannose also had an impact on macrophages by the suppression of IL-1β production [66]. Moreover, allulose has been reported as having inhibitory effects on pro-inflammatory adipokines and cytokines in diabetic mice [67]. Taken together, our study suggests that the underlying mechanisms of the anti-inflammatory activity of oligopeptide conjugation with mannose and allulose act by increasing cellular uptake and reducing inflammatory-related molecules.

Due to the range of significant bioactive activities, especially in the case of the antioxidant and anti-inflammatory activity of conjugated soybean oligopeptides [9], these conjugates might be candidates for functional ingredients for healthy foods, nutraceuticals, and cosmetics. As a rare sugar, allulose is generally regarded as safe by the FDA [68] and to date there have been no studies reporting the toxicity of mannose in humans [69,70]. In addition, there is evidence to suggest that soy protein, soy bioactive peptides, and soy isoflavones are generally safe for human consumption [71]. However, soy oligopeptides modified with either allulose (OT-AL) or mannose (OT-Man) are categorized as a new substance or a novel food; therefore, it is necessary to understand their potential risks and any adverse consequences of human exposure to verify their safety. This study assayed the cytotoxicity of OT-AL and OT-Man in various types of cell lines, which represent target organs in humans. The results show that OT-AL and OT-Man and their individual molecules were safe in the case of the liver (HepG2 and LX-2), kidney (HEK-293), and adipocytes (3T3-L1) (at up to 1000 µg/mL). Although OT, OT-AL, and OT-Man at high doses slightly decreased the cell viability of RAW 264.7 macrophages, these compounds did not show any toxicity to hPBMC viability (at up to 800 µg/mL), a non-proliferation cell type, suggesting that OT and their conjugated forms may exhibit inhibitory effects on cell proliferation rather than the induction of cell death. In addition, OT-AL and OT-Man (at up to 800 µg/mL) did not induce hemolysis in hRBCs. These results suggested that the conjugated peptide may be safe regarding the target organs and the cells in the blood circulation of the human body. Our data could be used to estimate starting doses for further acute oral toxicity tests in animal models to ensure their safety before studies in clinical trials. As OT-Man shows promising bioactivities to suppress LPS-induced inflammation, this conjugated form warrants further study for development as a functional ingredient for health products.

Genotoxicity assessment of OT, Man, and OT-Man was carried out using the Ames test in bacteria and SMART in *Drosophila*. In the Ames test, we screened the mutagenic activity of OT, Man, and OT-Man using the *S. typhimurium* strains TA98 and TA100 representative of a frameshift mutation and a base-pair substitution mutation, respectively, following the guidelines of OECD 471 [72]. 2-aminoanthracene (2-AA) and 2-(2-furyl)-3-(5-nitro-2-furyl)-acrylamide (AF-2) were used as positive controls in the test as commonly standard mutagens. 2-AA, aromatic amines, are most widely used as a pro-mutagen in the Ames test [29,30,73]. 2-AA requires metabolic activation through CYP1A1/2 and further catalysis into the metabolite that can bind to DNA resulting in the DNA adduct [74,75]. AF-2, an antimicrobial food additive, exerts carcinogenicity in mice [76]. OT, Man, and OT-Man (at non-toxic concentrations) did not induce the production of revertant colony numbers in both strains, TA 98 and TA 100, in the presence and absence of metabolic activation, suggesting that they have no potential pro-mutagenic or mutagenic impact. We also investigated the potential mutagenicity of OT, Man, and OT-Man using SMART in *Drosophila* as an alternative in vivo model. The mutagenicity of the sample can be detected by an abnormal hair phenotype on adult wings corresponding to two recessive wing cell marker genes; multiple wing hairs (*mwh*) and flare (*flr*) based on the loss of heterozygosity (LOH) caused by genetic damage during wing disc cells’ division in the larval stage [77]. Urethane, a known pro-mutagen, is metabolized by cytochrome P450 into an activate mutagen, vinyl carbamate epoxide, which can react with DNA as an alkylating agent resulting in deletion and point mutation [78]. Our data demonstrated that the mutant spots of all samples were not significantly different to the negative control, while urethane significantly induced mutagenesis. These results were consistent with the Ames test, adding weight to the evidence that no mutagenic effects of OT, Man, and OT-Man were observed in the *Drosophila*, suggesting their genome was safe following the application of the two test chemicals.

The limitations of this study which need to be borne in mind in further investigations are the need for the characterization of bioactive oligopeptides, especially their structure, amino acid sequences, the conjugation ratio between the sugar and the oligopeptides, functional properties (emulsion, solubility, foaming, and gelling), digestibility, and the bioavailability and bioaccessibility of the conjugated oligopeptides. In addition, to verify the safe use of the conjugated oligopeptides in humans, toxicity testing in animal models and in clinical trials needs to be carried out.

## 5. Conclusions

This study demonstrated a potential underlying mechanism of the anti-inflammatory activity of the formulated conjugated oligopeptides, especially in the case of OT-Man which may act via an increase in cellular uptake and the suppression of inflammation-related molecules. In addition, soybean oligopeptides, D-mannose, and D-mannose-conjugated oligopeptides present as safe for human use in terms of cytotoxicity and genome safety. This study provides potential information for health benefits and the safe use of conjugated soybean peptides as functional ingredients in health foods, nutraceuticals, and cosmetics.

## Figures and Tables

**Table 1:** Toxicity of oligopeptides, allulose and D-mannose on liver, kidney, macrophage, and adipocyte cell lines.

Cell Lines	Oligopeptide (OT) (µg/mL)		Allulose (AL) (µg/mL)		D-mannose (Man) (µg/mL)	
	IC_20_	IC_50_	IC_20_	IC_50_	IC_20_	IC_50_
HepG2	>1000	>1000	>1000	>1000	>1000	>1000
HEK293	>1000	>1000	>1000	>1000	>1000	>1000
LX-2	>1000	>1000	>1000	>1000	>1000	>1000
RAW 264.7	60	510	>1000	>1000	>1000	>1000
3T3-L1 Pre-adipocyte	>1000	>1000	>1000	>1000	>1000	>1000
3T3-L1 Mature-adipocytes	>1000	>1000	>1000	>1000	>1000	>1000

**Table 2:** Toxicity of oligopeptides conjugated with allulose and D-mannose on liver, kidney, macrophage, and adipocyte cell lines.

Cell Lines	Oligopeptide–Allulose (OT-AL) (µg/mL)		Oligopeptide–D-mannose (OT-Man) (µg/mL)	
	IC_20_	IC_50_	IC_20_	IC_50_
HepG2	>1000	>1000	>1000	>1000
HEK293	>1000	>1000	>1000	>1000
LX-2	>1000	>1000	>1000	>1000
RAW 264.7	270	>1000	450	>1000
3T3-L1 Pre-adipocyte	>1000	>1000	>1000	>1000
3T3-L1 Mature-adipocytes	>1000	>1000	>1000	>1000

**Table 3:** Toxicity of oligopeptides, allulose, D-mannose, and their oligopeptide-conjugated with allulose and D-mannose on peripheral blood mononuclear cell (PBMCs).

Extracts (µg/mL)	% Cell Viability (n = 5)
0	100
**Oligopeptide (OT)**	
100	114 ± 20.76
200	110 ± 23.32
400	117 ± 20.97
800	120 ± 24.60
**Allulose**	
800	108 ± 17.96
**Oligopeptide–Allulose (OT-AL)**	
100	104 ± 19.21
200	110 ± 22.12
400	111 ± 25.05
800	113 ± 24.14
**D-mannose**	
800	106 ± 13.26
**Oligopeptide–D-mannose (OT-Man)**	
100	99 ± 12.60
200	111 ± 20.32
400	114 ± 17.88
800	120 ± 16.60

**Table 4:** The hemolytic effect of oligopeptides, allulose, D-mannose, and the oligopeptide-conjugated with allulose and D-mannose.

Extracts (µg/mL)	% Hemolysis (n = 5)
**0.05% triton x-100**	100 ± 0.00
**Oligopeptide (OT)**	
100	1.98 ± 0.11
200	2.01 ± 0.16
400	1.76 ± 0.13
800	1.65 ± 0.27
**Allulose**	
800	1.76 ± 0.26
**Oligopeptide–Allulose (OT-AL)**	
100	2.19 ± 0.23
200	2.26 ± 0.16
400	2.56 ± 0.26
800	2.75 ± 0.37
**D-mannose**	
800	1.84 ± 0.42
**Oligopeptide–D-mannose (OT-Man)**	
100	2.14 ± 0.09
200	2.38 ± 0.12
400	2.54 ± 0.23
800	2.98 ± 0.32

0.05% triton x-100 was used as a positive control. The results are expressed as the mean ± SD, *n* = 5; 0–10% hemolysis is non-hemolysis, 10–25% hemolysis is slight hemolysis induction, and >25% hemolysis is high hemolysis induction.

**Table 5:** Mutagenicity of oligopeptide (OT), D-mannose (Man), and mannose-conjugated oligopeptide (OT-Man) in the *Salmonella typhimurium* strains TA100 and TA98 in the presence and absence of the S9 mix.

Treatment	Dose (per Plate)	His^+^ Revertant Colonies per Plate			
		TA100		TA98	
		+S9	−S9	+S9	−S9
DMSO	-	131.3 ± 11.0	122.8 ± 6.2	38.5 ± 3.8	30.7 ± 5.0
2-AA	0.5 µg	599.7 ± 8.7	-	325.8 ± 16.2	-
AF-2	0.01 µg	-	527.3 ± 15.0	-	-
AF-2	0.1 µg	-	-	-	293.0 ± 10.3
Oligopeptide (OT)	0.04 mg	133.3 ± 6.7	128.2 ± 4.5	34.8 ± 6.5	30.2 ± 1.8
	0.2 mg	144.0 ± 10.0	131.2 ± 3.8	38.2 ± 5.8	31.3 ± 7.0
	1 mg	161.5 ± 6.5	147.5 ± 7.8	44.3 ± 6.3	34.5 ± 4.8
	5 mg	228.2 ± 8.8 (k)	191.8 ± 0.2 (k)	62.8 ± 9.8 (k)	43.5 ± 1.8 (k)
D-Mannose (Man)	0.04 mg	127.5 ± 2.5	135.2 ± 5.2	37.7 ± 5.7	30.5 ± 2.5
	0.2 mg	140.3 ± 2.3	137.5 ± 4.5	37.8 ± 3.8	30.8 ± 4.8
	1 mg	139.0 ± 0.7	135.2 ± 4.5	36.5 ± 7.5	27.8 ± 6.5
	5 mg	138.5 ± 4.2	129.7 ± 4.7	38.5 ± 7.8	28.7 ± 6.0
Mannose-conjugated oligopeptide (OT-Man)	0.04 mg	139.5 ± 7.2	136.0 ± 5.7	37.5 ± 4.8	28.8 ± 6.2
	0.2 mg	137.3 ± 4.0	128.2 ± 5.2	39.3 ± 9.0	29.0 ± 4.7
	1 mg	145.2 ± 4.8	127.2 ± 1.8	41.2 ± 8.2	32.5 ± 4.5
	5 mg	146.3 ± 5.0 (k)	140.8 ± 9.2 (k)	45.0 ± 8.7	31.3 ± 4.7

Values are represented as the mean ± SEM. k; killing effect.

**Table 6:** Frequency of mutant spots on the wings of *Drosophila* with high bioactivation.

Samples		Number of Wings	Frequency of Mutant Spots per Individual (Number of Spots) ^#^			
			Small Single (1–2 Cells)	Large Single (>2 Cells)	Twin	Total Spots
DI (negative control)		40	0.63 (25)	0.05 (2)	0.00 (0)	0.68 (27)
20 mM urethane		40	6.10 (244) +	3.40 (136) +	0.68 (27) +	10.18 (407) +
OT	62.5 µg/mL	40	0.50 (20) −	0.00 (0) −	0.00 (0) −	0.50 (20)−
125 µg/mL		40	0.50 (20) −	0.00 (0) −	0.00 (0) −	0.50 (20) −
250 µg/mL		40	0.28 (11) −	0.00 (0) −	0.00 (0) −	0.28 (11) −
500 µg/mL		40	0.28 (11) −	0.03 (1) i	0.00 (0) −	0.30 (12) −
1000 µg/mL		40	0.43 (17) −	0.00 (0) −	0.00 (0) −	0.43 (17) −
2000 µg/mL		40	0.45 (18) −	0.03 (1) i	0.00 (0) −	0.48 (19) −
5000 µg/mL		40	0.35 (14) −	0.03 (1) i	0.00 (0) −	0.38 (15) −
Man	62.5 µg/mL	40	0.40 (16) −	0.03 (1) i	0.00 (0) −	0.43 (17)-
125 µg/mL		40	0.50 (20) −	0.03 (1) i	0.00 (0) −	0.53 (21) −
250 µg/mL		40	0.43 (17) −	0.03 (1) i	0.00 (0) −	0.45 (18) −
500 µg/mL		40	0.25 (10) −	0.03 (1) i	0.00 (0) −	0.28 (11) −
1000 µg/mL		40	0.25 (10) −	0.03 (1) i	0.00 (0) −	0.28 (11) −
2000 µg/mL		40	0.23 (9) −	0.00 (0) −	0.00 (0) −	0.23 (9) −
5000 µg/mL		40	0.18 (7) −	0.00 (0) −	0.00 (0) −	0.18 (7) −
OT-Man	62.5 µg/mL	40	0.25 (10) −	0.00 (0) −	0.00 (0) −	0.25 (10) −
125 µg/mL		40	0.25 (10) −	0.00 (0) −	0.00 (0) −	0.25 (10) −
250 µg/mL		40	0.30 (12) −	0.00 (0) −	0.00 (0) −	0.30 (12) −
500 µg/mL		40	0.25 (10) −	0.00 (0) −	0.00 (0) −	0.25 (10) −
1000 µg/mL		40	0.43 (17) −	0.00 (0) −	0.00 (0) −	0.43 (17) −
2000 µg/mL		40	0.13 (5) −	0.00 (0) −	0.00 (0) −	0.13 (5) −
5000 µg/mL		40	0.18 (7)−	0.00 (0)−	0.00 (0)−	0.18 (7)−

^#^ Statistical diagnoses using estimation of spot frequencies and confidence limits in comparison with DI (negative control); + = positive; − = negative; i = inconclusive. Probability levels: α = β = 0.05. The one-sided statistical test “m” is an increased mutation frequency compared with the spontaneous frequency (m times).

## Data Availability

The original contributions presented in the study are included in the article, further inquiries can be directed to the corresponding author.
